# Interplay between structural deformations and flat band phenomenology in twisted bilayer antimonene†

**DOI:** 10.1039/d1ra05301a

**Published:** 2021-08-17

**Authors:** Alan C. R. Souza, Matheus J. S. Matos, Mario S. C. Mazzoni

**Affiliations:** Departamento de Física, Universidade Federal de Minas Gerais Belo Horizonte MG Brazil 31270-901 mazzoni@fisica.ufmg.br; Departamento de Física, Universidade Federal de Ouro Preto Ouro Preto MG Brazil 35400-000

## Abstract

In this work we apply first principles calculations to investigate the flat band phenomenology in twisted antimonene bilayer. We show that the relatively strong interlayer interactions which characterize this compound have profound effects in the emergence and properties of the flat bands. Specifically, when the moiré length becomes large enough to create well defined stacking patterns along the structure, out-of-plane displacements take place and are stabilized in the regions dominated by the AB stacking, leading to the emergence of flat bands. The interplay between structural and electronic properties allows for detection of flat bands in higher twist angles comparable to other two-dimensional materials. We also show that their energy position may be modulated by noncovalent functionalization with electron acceptor molecules.

## Abstract 2

Strong interlayer interactions allows for electron localization and emergence of flat bands in relatively higher twist angles in Sb bilayer. Noncovalent functionalization with electron acceptor molecules may modulate their energy position.

## Graphical abstract

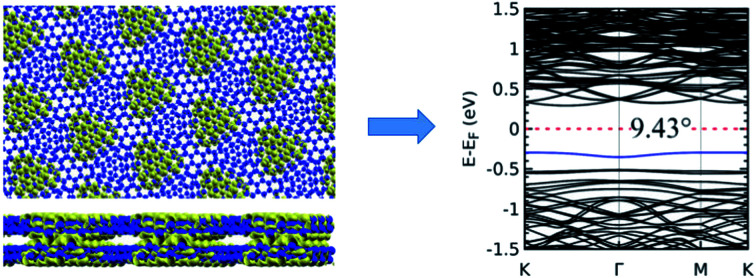

## 1. Introduction

The experimental observation of unconventional superconductivity in twisted bilayers graphene (TBG)^1^ established a new milestone in the investigation of two-dimensional (2D) materials. After that, observation of insulating states,^2^ magnetism,^3^ and optically induced flat bands^4^ showed that rotated multilayer graphene structures are a rich playground for fundamental physics, in which the existence of Bloch flat bands plays^5^ a crucial role. One of the most important aspects of these findings is the strong rotation angle dependence, which is behind the existence of the so-called “magic angles”^6^ for the observation of TBGs exotic properties. In these angles, the interlayer interactions become strong and the competition between kinetic and Coulomb potential changes the density of states giving rise to flat bands in the vicinity of the Fermi level. In addition, results for twisted double bilayer graphene (tDBG)^7^ showed a strong stacking dependence of the flat bands in angles slightly higher than 1.1°, which suggests that it is possible to “functionalize” the TBG with controlled incorporation of 2D layers.^8^ Besides graphene, other 2D van der Waals materials, such as hexagonal boron nitride (hBN), can also present interesting electronic modulation due to layer rotation. Indeed, theoretical results have shown the emergence of flat Bloch bands in twisted bilayer hBN without the magic angle mechanism.^9^ In this case, different regions of moiré superlattice are characterized by different stacking patterns and, if the twisted angle is small enough, electronic states arising from these patterns could fall into the insulator gap and form non-dispersive bands with spatially localized wavefunctions. A common feature in all these systems is that the phenomenology occurs only for very small angles – 1.1° for TBG, less than 2.13° for tDTGB, and less than 3° for hBN. Consequently, the theoretical descriptions of these states rely on tight-binding approaches or continuous models^10–15^ which give important insights into the electronic structure and allow for the exploitation of its angular dependence.

There is yet another class of 2D materials in which the idiosyncrasies of the twisted configurations may show up with important particularities. The beta phase honeycomb structures of the pnictogens As, Sb, and Bi are characterized by relatively stronger interlayer interactions, which classify these materials as pseudolayered compounds.^16^ May these interactions drive localized out-of-plane deformations in rotated structures with separated regions of well defined stacking patterns? If this is the case, is it possible to establish an interplay between geometric distortions and electronic localization underlying the flat band phenomenology in these materials?

In this paper, we address these questions by performing density functional theory (DFT) calculations for moiré superlattices of antimonene bilayers twisted by 21.79°, 13.17°, 9.43°, and 6.01°. Our calculations showed that, indeed, the twist highly modulates the electronic structure: we detected the flat band phenomenology for all angles, with an increasing degree of localization as the twist angle decreases, with well defined regions with AA, AB and AC patterns. Importantly, we found that the interlayer distance and the degree of out-of-plane distortions are modulated along the bilayer and may be used as fingerprints of the stacking patterns. The flat bands are found to be highly localized in the AB stacking regions, which correspond to the smallest interlayer distance and greater distortions. Lastly, we showed that non-covalent functionalization with an electron acceptor molecule is able to shift the energy of the flat band state, positioning it close to the Fermi level.

## 2. Methods

We employed the SIESTA^17^ implementation of the pseudopotential spin DFT formalism,^18,19^ expanding the Kohn–Sham eigenfunctions in a finite range double-ζ basis set augmented by polarization functions (DZP basis set). We made use of the Gradient Generalized Approximation (GGA) within the Perdew–Burke–Ernzerhof (PBE) parametrization^20^ for the exchange–correlation functional. We employed a mesh cutoff of 450 Ry to define the grid for real space integrations, and the geometries were considered relaxed when the maximum force component in any atom was less than 10 meV Å^−1^. To sample the Brillouin zone, we defined a *k*-grid using the Monkhorst–Pack scheme with 3 × 3 × 1, 7 × 7 × 1, 12 × 12 × 1 and 15 × 15 × 1 meshes for the 6.01°, 9.43°, 13.17°, and 21.79° structures, respectively. The Cartesian coordinates and lattice vectors of all structures investigated in this work can be found in the ESI.†

## 3. Results and discussion

**Fig. 1:**
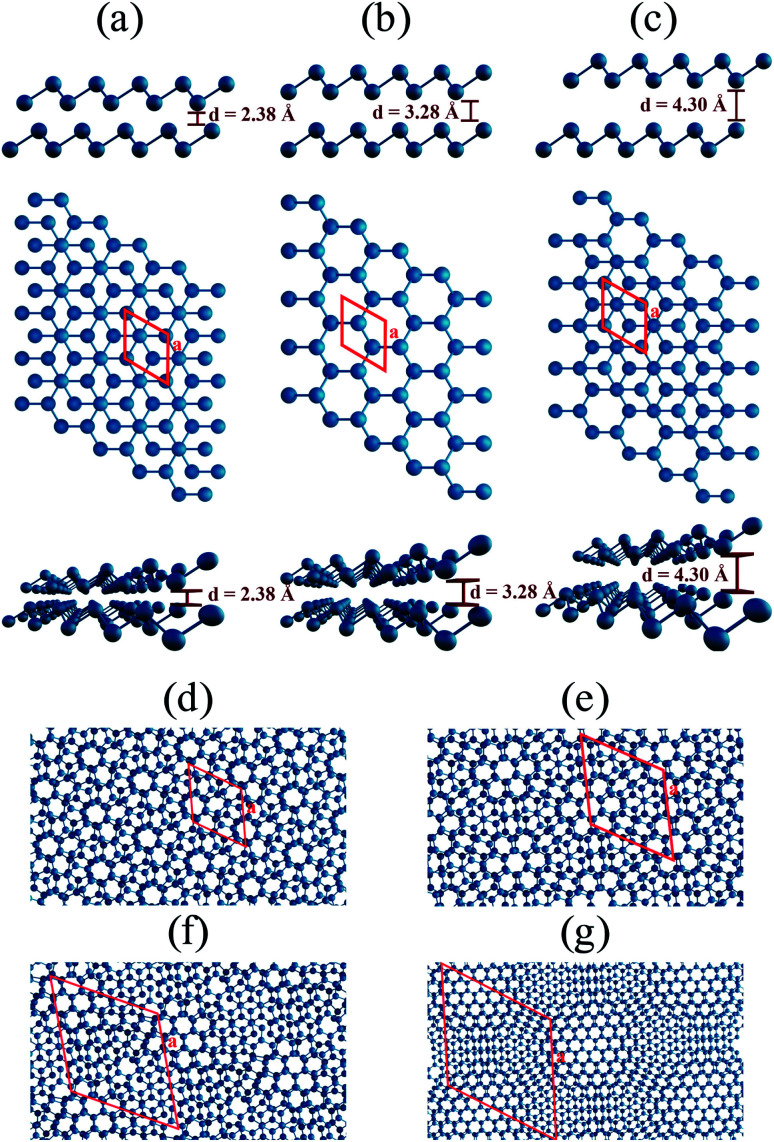
Bilayer antimonene: optimized structures for (a) AB, (b) AA and (c) AC stacking orders in side view (upper panel), top view (middle panel) and in perspective (bottom panel). Top views of the relaxed rotated bilayer antimonene structures with twist angles of (d) 21.79°, (e) 13.17°, (f) 9.43°, and (g) 6.01°. In the top views, the primitive cells are indicated by red lines. The lattice vectors of the twisted configurations, as well as their Cartesian coordinates, can be found in the ESI.†

Antimony, in its bulk beta-phase, is a layered compound characterized by a hexagonal symmetry. Facile methods to produce its few-layer counterparts have been reported,^21^ paving the way for their manipulation at nanoscale. Concerning bilayers, Fig. 1(a)–(c) show top and side views of structures built upon the beta-phase in three stacking orders, AB, AA and AC, respectively. The AC stacking may be constructed from the AB by translating the top layer of two-thirds and one-third in the direction of each lattice vector, respectively. The same procedure in the bottom layer leads to the AA stacking. Important to the reasoning we shall pursue in this work, the relative orientation of the atoms in different layers, as the side views in Fig. 1 clearly show, implies a strong dependence of the interlayer distance with the stacking order: 2.38, 3.28, and 4.30 Å for AB, AA, and AC, respectively, as determined by DFT calculations within our implementation scheme, which also indicate the AB as the energetically most favorable bilayer stacking, followed by AA and AC (0.018 and 0.039 eV per atom relative to AB, respectively). Therefore, in our models, we started from the AB stacking, and we built four moiré superlattices with twist angles of 21.79°, 13.17°, 9.43°, and 6.01°. The models comprise 28, 76, 148 and 364 atoms, respectively. For each of them, we performed a full geometry optimization. We show in Fig. 1(d)–(g) the top views of the resulting structures, which allow for a clear identification of AA domains. The distance between these domains constitute the moiré length – we found 10.9, 17.9, 25.1, and 39.5 Å in each case, respectively.

**Fig. 2:**
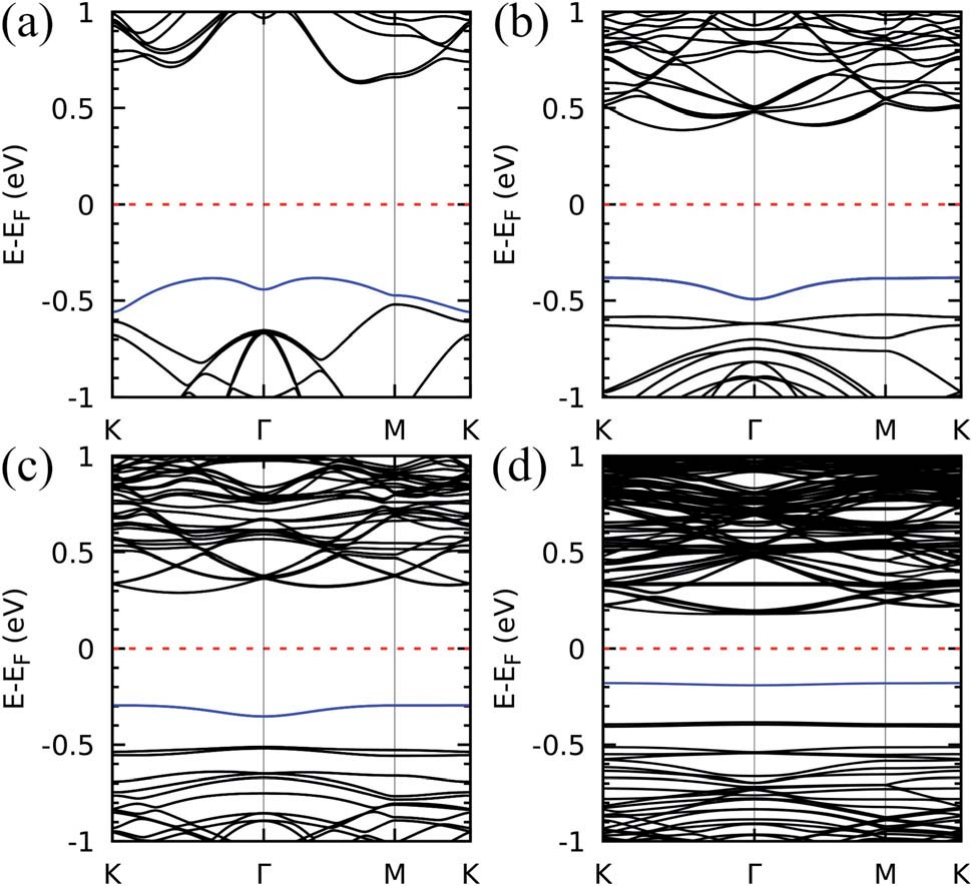
The flat band emergence: band structure for the twist angles (a) 21.79°, (b) 13.17°, (c) 9.43°, and (d) 6.01°. The valence band maximum is highlighted in blue for visualization purposes. The Fermi level is set to zero.

Next, we characterized the electron states for each moiré superlattice by determining the DFT band structures, as shown in Fig. 2. The semiconducting behaviour with indirect energy gap is a common feature. A sizable reduction of the bandgap upon decreasing twist angle is clearly seen – the energy difference between the bottom of the conduction band and the occupied band indicated by a blue curve in the figure varies from ∼1.0 eV (21.79°) to ∼0.4 eV (6.01°). Concerning the flat band formation, the effect of the twist angle shows up as a notable modulation of the valence band maximum (VBM, highlighted in blue in all figures) from dispersive (left panel, with 21.79°) to completely flat (right panel, with 6.01°). In this respect, the results are similar to those reported for the other pnictogens, phosphorous and arsenic, both beta-phase bilayers.^22,23^Fig. 2 indicates that the band evolution can be addressed in three steps: (I) the VBM for 21.79° is still slightly dispersive and mixed with other valence bands (Fig. 2(a)); (II) for 13.17°, a great decrease in the VBM band dispersion leads to quasi-flat isolated bands in the proximity of the other valence bands (Fig. 2(b)); (III) for 9.43° and 6.01° (Fig. 2(c) and (d), respectively), well-defined dispersionless bands within the bandgap are clearly distinguished in the band structures.

This is interesting since unconventional superconductivity^1^ and insulating strongly correlated states^2^ are ascribed to the existence of flat bands below the Fermi level in twisted structures, but only for very small magic angles. The emergence of flat bands in the relatively greater twist angles shown in Fig. 2 is mostly due to two important physical aspects of antimonene which we shall discuss: the strong interlayer interactions and the atomic displacements in the different stacking patterns across the superlattice.

**Fig. 3:**
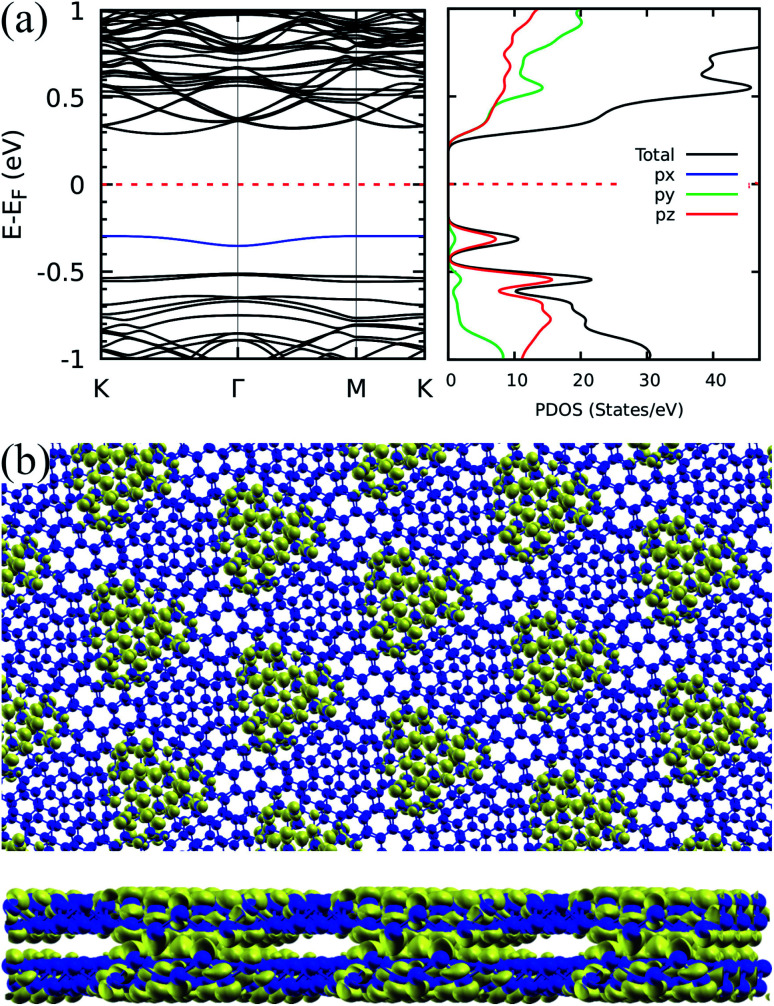
Orbital character and localization for the 9.43° case: (a) band structure for the bilayer antimonene twisted in 9.43° and projected density of states. (b) Top and side views of the corresponding relaxed structure (antimony atoms represented by blue spheres) superimposed to the isosurface plot of the charge density associated to the flat band highlighted in blue in the band structure. The isosurface value is of 0.00015 electron per bohr^3^.

To pursue a further understanding on the nature of the flat bands, we reproduce in Fig. 3(a) the band structure for the 9.43° twisted Sb bilayer, presenting also the projected density of states (PDOS). The predominant contribution to the flat bands comes from p_*z*_ orbitals due to the expected hybridization scheme in hexagonal structures and the consequent role of the alignment of p_*z*_ orbitals in the interlayer orbital overlap, which defines the strength of interlayer interactions.

**Fig. 4:**
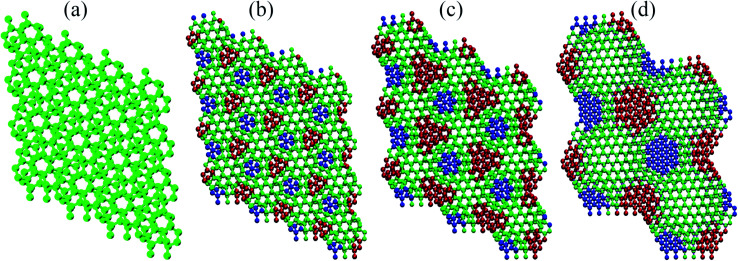
Stacking patterns in Sb superlattices: from left to right, the relaxed geometries for antimonene bilayers twisted in (a) 21.79°, (b) 13.17°, (c) 9.43°, and (d) 6.01°. Green, blue, and red spheres represent atoms with vertical distances typical of those of the AA, AC, and AB stacking configurations, respectively.

This points out to the importance of geometric aspects in defining these interactions. To characterize these aspects in greater detail, we first show that, similar to the phenomenology reported for hBN,^9^ the electronic density associated to the flat bands is well localized in real space. Indeed, Fig. 3(b) presents an electron density isosurface plot corresponding to the flat band (indicated by the blue curve in Fig. 3(a)), clearly showing its localized character, which, as we shall show, is related to well separated AB stacking patterns within the superlattice. Moreover, the bottom panel of Fig. 3(b) suggests a crucial peculiarity of the pnictogen case: out-of-plane displacements for small angles in the vicinity of the AB stacking patterns, which enhances the electronic density in the interlayer region. The phenomenology can be clearly visualized in Fig. 4, and relies on the fact that, in the lowest energy AB configuration of the Sb bilayer, the interlayer distance is smaller than those of the other stackings (2.38, 3.28, and 4.30 Å for AB, AA and AC, respectively, within our DFT implementation). Fig. 4(a)–(d) show the four twisted configurations in a representation in which the green, blue and red spheres represent Sb atoms with interlayer distances characteristic of the AA, AB and AC stackings, respectively. For large twist angles, as shown in Fig. 4(a) for 21.79°, the moiré length is small and the stacking patterns are poorly defined. As a consequence, the interlayer distance is rather uniform along the structure and matches that of the AA pattern. On the other hand, when the moiré length is high enough to create and isolate well defined regions with AB, AA and AC patterns, as shown in Fig. 4(b)–(d) for 13.17°, 9.43°, 6.01°, respectively, out-of-plane displacements may be stabilized in the AB regions. This must have profound consequences in the tunneling between layers, which is associated with flat bands.^6^

**Fig. 5:**
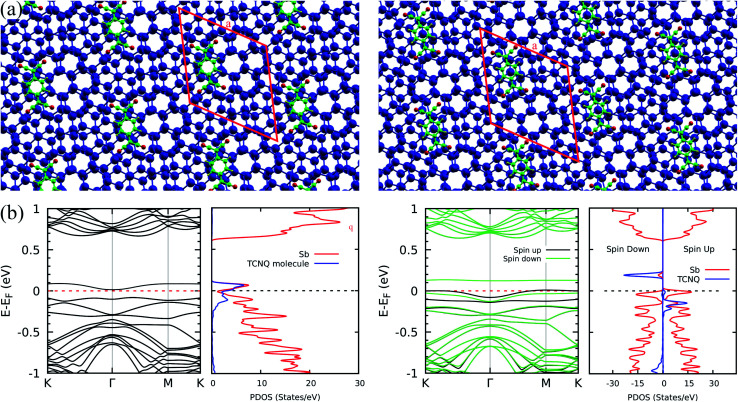
The Sb + TCNQ case: (a) relaxed structures for the 13.17°, twisted antimonene bilayer with the TCNQ molecule initially adsorbed onto the AB (left) and AC (right) regions. The blue, green, red, and yellow spheres represents the Sb, C, N and H atoms respectively. (b) Band structure and projected density of states for the AB (left) and AC (right) cases. The electronic states shown in the band structure on the left are not spin polarized. In the band structure shown on the right, green and black curves represent the two spin components. The blue and red curves of the PDOS plots represent projections on the TCNQ molecule and on the Sb atoms, respectively.

Once the flat band phenomenology has been established, a question arises on the possible modulation of its energy position. A possible strategy may be based on non-covalent functionalization with electron acceptor molecules. In fact, Abellán G. *et al.*^24^ have reported the efficiency of this mechanism in tailoring the electronic properties of few layer antimonene through formation of stable complexes involving, for instance, tetracyanoquinodimethane (TCNQ – C_12_H_4_N_4_) molecules. Therefore, we performed calculations for TCNQ interacting with bilayer antimonene twisted by 13.17°. We considered adsorption onto sites with stacking patterns characteristic of the AA, AB and AC configurations. Fig. 5(a) and (b) show, respectively, the relaxed geometries (upper panels) and band structures with the corresponding projected density of states (bottom panels) for the AB (left) and AC (right) cases. We omitted the AA electronic characterization since it is very similar to the AB case. For the AB case, the TCNQ molecule shifts to a region between the AB and AA patterns during the geometric optimization, as shown in the left panel of Fig. 5(a). For all relative positions, we verified the strong charge transfer nature of the interaction, which defines the low energy features of the spectra. In the AC configuration, shown in the right part of the figure, this brings an antimonene flat band to the Fermi level, while the states associated to the molecule become spin polarized and can be clearly identified above and below the Fermi level. For the other configurations, as shown on the left of Fig. 5(a) for the AB case, flat states are found at energies ±0.1 eV from the Fermi level, and present a high degree of hybridization between antimonene and the molecule. Adsorption on the AC pattern is energetically more favourable by 0.044 and 0.127 eV when compared to AB and AA patterns, respectively. This phenomenology is not restricted to a specific twist angle: we observed similar trends – the shift of flat states to the Fermi level – in calculations performed for a twist angle of 6.01°.

## 4. Conclusions

In conclusion, through first principles calculations, we demonstrated a rich interplay between structural and electronic aspects involved in flat band formation in twisted antimonene bilayers. The phenomenology has similar aspects to those observed in polar compounds, such as hBN, with well separated stacking patterns determining the localization of flat bands. However, due to strong interlayer interactions in pnictogen compounds, which greatly distinguishes interlayer distances in different stacking patterns, the process in Sb bilayer involves large out-of-plane deformations, which, in turn, allows for electron localization and emergence of flat bands in relatively higher twist angles when compared to other 2D material. Moreover, using well-established techniques for noncovalent functionalization with electron acceptor molecules, it is possible to modulate the energy position of the flat bands, positioning them close to the Fermi level. Altogether, our work reinforces the idea that antimonene is an important material for the exploitation of fundamental physical phenomena, such as strongly correlated insulating states and superconductivity.

## Conflicts of interest

There are no conflicts to declare.

## Supplementary Material

RA-011-D1RA05301A-s001
