# Supplementary material for: Interplay between structural deformations and flat band phenomenology in twisted bilayer antimonene
Source: RSC Adv. 2021 Aug 17;11(45):27855–9. doi: 10.1039/d1ra05301a (PMC9038057; doi:10.1039/d1ra05301a)
Supplement: RA-011-D1RA05301A-s001 [file RA-011-D1RA05301A-s001.pdf]

Electronic Supplementary Material (ESI) for RSC Advances.
This journal is © The Royal Society of Chemistry 2021

Electronic Supplementary Information (ESI)

This file contains the lattice vectors and cartesian coordinates (in Bohr) for the following systems (all fully converged);

21.79° Twisted bi-layer
13.17° Twisted bi-layer
13.17° Twisted bi-layer + TCNQ molecule (AC)
13.17° Twisted bi-layer + TCNQ molecule (AB)
9.43° Twisted bi-layer
6.01° Twisted bi-layer

################# POSITIONS AND LATTICE VECTORS IN BOHR#################

```
#   21.79°  Twisted Angle :
#   Lattice Vectors (Cartesian, Bohr):
       -1.696152242      20.567087540       0.000000000
      -18.660441678       8.814237699       0.000000000
        0.000000000       0.000000000      46.145716919
        28 # Number of atoms
#Cartesian Coordinates: chemical_specie_label, atomic_number, coordinates
  1   51     -2.982866464      9.575866420     -2.745189850
  1   51     -3.402283927      5.007077511      0.268880567
  1   51     -7.529839693      3.240279552     -2.895345097
  1   51     -8.202213809     -1.175147424      0.298982370
  1   51      4.706925578      8.746293714     -2.903678126
  1   51      4.300377663      4.312649998      0.301237927
  1   51      0.256208386      2.390390789     -2.863634582
  1   51     -0.247279354     -1.942235953      0.462234647
  1   51     -4.384691011     -3.866156429     -2.589193210
  1   51     -4.884526654     -8.392126935      0.477414805
  1   51      8.010436323      1.745765942     -2.825122919
  1   51      7.506664913     -2.818965836      0.176936405
  1   51      3.256914163     -4.666697128     -2.768837155
  1   51      2.865381586     -9.172868937      0.341971554
  1   51     -9.879714517     12.668642517      7.101388693
  1   51     -8.549362745      8.456627074     10.362877271
  1   51    -11.662175719      5.107106589      7.318626138
  1   51    -10.434208061      0.727426097     10.394655006
  1   51     -2.194751487     14.779331264      6.904669616
  1   51     -0.966083303     10.565099521     10.194797897
  1   51     -4.201515473      7.285173062      7.218611622
  1   51     -2.913993158      2.983527178     10.370222326
  1   51     -6.075431761     -0.171868034      7.186424308
  1   51     -4.853733147     -4.688657010     10.060093892
  1   51      3.434542979      9.291331610      7.169561809
  1   51      4.532382826      4.982928345     10.377426033
  1   51      1.382672721      1.878472616      7.139305762
  1   51      2.468560290     -2.551929579     10.194331867
------------------------------------------------------------------------------------------
```

13.17°

	-3.902803402	33.783108929	0.000000000	
	-31.209351507	13.514751566	0.000000000	
	0.000000000	0.000000000	48.234830549	
	76			
1	51	-7.776608065	15.821560133	-2.298708920
1	51	-7.744610332	10.989698066	0.306352164
1	51	-11.625671911	9.028734235	-3.047757333
1	51	-11.756788367	4.485585976	0.021901497
1	51	-15.542956045	2.140984199	-3.195616528
1	51	-15.821421800	-2.140206651	0.231913757
1	51	-0.135805673	15.796076081	-2.857314097
1	51	-0.043881628	11.225322370	0.196296081
1	51	-3.878887273	8.948065246	-2.991920996
1	51	-3.840026770	4.485862437	0.192440233
1	51	-7.749334246	2.103302217	-2.852806352
1	51	-7.848953822	-2.071436532	0.728753042
1	51	-11.650578968	-4.752804855	-2.201127460
1	51	-11.986323214	-9.039340663	1.247970540
1	51	7.784396643	15.695587960	-3.005470632
1	51	7.542941924	11.355030172	0.352104145
1	51	3.859471068	9.034134229	-2.968581489
1	51	3.907057089	4.566388107	0.206438527
1	51	0.063134018	2.292182932	-2.972210520
1	51	0.212814578	-2.046845951	0.368189512
1	51	-3.942593531	-4.514401691	-2.238910268
1	51	-3.780191415	-8.749695222	1.273875498
1	51	-7.791075133	-11.249658056	-1.536870095
1	51	-7.622671152	-16.002935853	1.224723121
1	51	11.740050438	8.972651118	-2.270206987
1	51	11.584736553	4.375418081	0.711066506
1	51	7.912432046	2.372101829	-2.854655526
1	51	7.872581703	-2.280170353	0.039113891
1	51	3.840012832	-4.447298286	-2.982531318
1	51	4.105925395	-8.864516733	0.259741296
1	51	-0.235152783	-11.176111125	-2.179742718
1	51	0.182571832	-15.832632813	0.713036037
1	51	15.802763540	2.425616652	-2.229251190
1	51	15.635680429	-2.495076052	0.201709529
1	51	11.770476506	-4.395948823	-3.204062038
1	51	11.715043776	-8.998733151	-0.201989246
1	51	7.672921101	-11.244644436	-3.171661623
1	51	7.807898654	-15.695803903	0.047074431
1	51	-15.233450789	19.391868877	6.244027208
1	51	-13.924872413	15.302299420	9.698973855
1	51	-17.382586122	11.812788274	7.246740638
1	51	-16.147528187	7.704433521	10.675753761
1	51	-19.460990226	4.495627315	7.687053726
1	51	-18.335110792	0.021872652	10.674010386
1	51	-7.180759964	20.972808122	6.249981482
1	51	-6.371550098	16.828708833	9.782562049
1	51	-9.596646029	13.561271683	6.788278841
1	51	-8.563637615	9.502313217	10.350681718

1	51	-11.927973626	6.293264264	7.450369400
1	51	-10.753806004	1.874652084	10.481024385
1	51	-14.161268127	-0.953878155	7.241918802
1	51	-12.869551978	-5.698637219	9.681004273
1	51	0.482019414	22.877526597	7.256951144
1	51	1.246737740	18.530988053	10.516265463
1	51	-1.763606939	15.390331751	7.165467600
1	51	-0.929092367	11.104609738	10.470924403
1	51	-4.221006301	8.088326184	7.290738314
1	51	-3.243645293	3.720004071	10.456047599
1	51	-6.525140410	0.871763746	7.123622806
1	51	-5.463901945	-3.849232540	9.722434417
1	51	-8.679340217	-6.619737645	6.218646387
1	51	-7.766694556	-11.261064049	9.023521690
1	51	5.638365616	17.335122044	7.457468823
1	51	6.743694521	12.814939047	10.346349599
1	51	3.333964141	9.772527407	7.290804697
1	51	4.302640371	5.400855972	10.455271781
1	51	1.013876587	2.386640194	7.271230683
1	51	1.958359568	-2.092599374	10.316917680
1	51	-1.015998956	-5.025933180	6.742113820
1	51	-0.372947037	-9.626216710	9.684001106
1	51	10.766645801	11.694782718	6.767254789
1	51	11.973338544	7.262852068	9.756869858
1	51	8.424314098	3.996288800	7.133775710
1	51	9.659050900	-0.183140173	10.476258863
1	51	6.427382532	-3.402175105	7.429786342
1	51	7.310195412	-7.752006351	10.669438605

13.17º + TCNQ (AC)

-3.931264675	33.866073422	0.000000000
-31.282425990	13.565905513	0.000000000
0.000000000	0.000000000	54.412840971
96		

1	51	-7.284482645	16.091466047	-1.739546578
1	51	-7.122308020	11.365658257	1.062083848
1	51	-11.021449816	9.477934533	-2.342729705
1	51	-11.200849893	4.587698382	0.145142684
1	51	-15.045344097	2.641116876	-3.274029857
1	51	-15.112505108	-1.914495718	-0.192248969
1	51	0.330856590	16.191617023	-2.301075868
1	51	0.683417668	11.550120170	0.630289858
1	51	-3.328089509	9.323009200	-2.359392877
1	51	-3.294742407	4.526664034	0.308937637
1	51	-7.198597817	2.556947578	-3.023543302
1	51	-7.357271042	-1.999365832	0.028686568
1	51	-11.176192606	-4.349546665	-3.161201136
1	51	-11.430902087	-8.615974591	0.299817147
1	51	8.191760570	16.093844590	-3.120584071
1	51	8.289022155	11.653584049	0.134355508
1	51	4.326099493	9.298641569	-2.823699669
1	51	4.383916299	4.740981016	0.254828947

1	51	0.579898772	2.468151849	-2.981810509
1	51	0.621380597	-2.018725152	0.165267730
1	51	-3.336254373	-4.385363993	-2.839043428
1	51	-3.446138257	-8.586208320	0.720101052
1	51	-7.288106223	-11.192358104	-2.227422313
1	51	-7.527274124	-15.583152826	1.082286368
1	51	12.181471628	9.249074846	-2.893839593
1	51	12.019740745	4.869874378	0.422488819
1	51	8.314448770	2.598590394	-2.921191389
1	51	8.365793560	-1.937025074	0.158797496
1	51	4.514887108	-4.119204391	-3.086130722
1	51	4.646420980	-8.487407768	0.234159051
1	51	0.456743074	-10.956705681	-2.310183415
1	51	0.642799114	-15.288010596	1.096503666
1	51	16.125068524	2.510724376	-2.358984592
1	51	16.086393941	-2.086015019	0.623643632
1	51	12.385121302	-4.089102315	-2.922315245
1	51	12.334769465	-8.761821280	-0.056014911
1	51	8.291819505	-10.917817834	-3.075244001
1	51	8.520277093	-15.311096222	0.218817838
1	51	-15.771138841	18.664392670	6.610614511
1	51	-14.643533644	14.273782884	9.681689355
1	51	-18.166646946	10.962874757	7.084690271
1	51	-16.963210203	6.748398288	10.394170094
1	51	-20.118609331	3.604925571	7.185198076
1	51	-19.287200413	-0.839591098	10.315362229
1	51	-8.029022473	20.585601557	5.894148086
1	51	-7.010814643	16.009310145	8.764218387
1	51	-10.648289906	12.778690429	6.219062251
1	51	-9.267561561	8.736461212	9.704608210
1	51	-12.763682862	5.312875592	7.168316427
1	51	-11.469931510	1.105285282	10.454961068
1	51	-14.772503562	-2.081027860	7.430912636
1	51	-13.719529194	-6.587679101	10.404303923
1	51	-0.157459935	22.199052857	6.241851660
1	51	0.452301168	17.588768883	9.168192465
1	51	-2.612377483	14.430580153	5.859450465
1	51	-1.630029590	10.198174625	9.227456551
1	51	-5.136824429	7.024403458	6.486223105
1	51	-3.869728958	2.952723573	9.939658351
1	51	-7.301516601	-0.264102890	7.134634260
1	51	-6.075690303	-4.610160340	10.303252248
1	51	-9.438595697	-7.528843458	7.106065668
1	51	-8.164313713	-12.294365572	9.508853617
1	51	5.246957738	16.261730267	6.844340309
1	51	5.956495867	11.916090361	10.133177324
1	51	3.010287105	8.839338540	6.637002704
1	51	3.700733298	4.579898336	10.013383255
1	51	0.444752056	1.503402119	6.849321359
1	51	1.389674362	-2.747322047	10.241977785
1	51	-1.871864704	-5.621178451	6.880935036
1	51	-0.700848625	-10.488078071	9.165712388

1	51	10.394735838	10.766976560	7.069462685
1	51	11.391422348	6.274269577	10.042569083
1	51	8.049837918	3.128560027	7.008979297
1	51	8.943877948	-1.156996945	10.322663071
1	51	5.672639492	-4.188596376	7.135685761
1	51	6.605991107	-8.787629893	10.013446563
2	6	-1.679693489	20.579237109	16.142148804
2	6	-4.259578979	21.392472179	16.012138645
2	6	-6.184649317	19.486060138	16.015276356
2	6	-5.567431716	16.936307862	15.962972910
2	6	-2.981080750	16.123228719	15.909952162
2	6	-1.059577718	18.029062668	16.089718379
2	6	-2.337371306	13.492664470	15.600137996
2	6	-4.897587050	24.031968202	15.732321965
2	6	0.217316723	12.691270515	15.349712686
2	6	-3.002942852	25.905549968	15.373014689
2	6	-4.226106863	11.608634554	15.251638663
2	6	-7.437022658	24.815084112	15.329453231
3	7	-5.810117556	10.054781546	14.907337210
3	7	-1.412715715	27.437194294	14.952862366
4	1	-7.078486852	15.498493452	15.932022841
4	1	-0.168319558	22.014749315	16.234311462
3	7	2.356367222	12.050970528	15.106168516
3	7	-9.550564606	25.435579440	14.884004651
4	1	-8.187157157	20.071128688	16.004318591
4	1	0.940901075	17.441898970	16.164537461

13.17 + TCNQ (AB - FINAL)

-3.881548768	33.856616057	0.000000000
-31.196898511	13.525981887	0.000000000
0.000000000	0.000000000	54.412840971
96		

1	51	-8.136577325	15.263999664	-2.815384012
1	51	-7.964248634	10.682991437	0.232258330
1	51	-11.851189583	8.413213699	-2.903300063
1	51	-11.890882318	3.900827293	0.224588581
1	51	-15.588530362	1.486052871	-3.058815626
1	51	-16.014552961	-2.736491047	0.446211954
1	51	-0.295811478	15.142933533	-3.445532101
1	51	-0.335841524	10.881364515	0.028653626
1	51	-4.165126058	8.435020768	-3.029003729
1	51	-4.005854798	4.092416483	0.331704534
1	51	-7.849115880	1.711161962	-2.766190302
1	51	-7.805754770	-2.564630767	0.684191365
1	51	-11.794380428	-5.121971383	-2.083072393
1	51	-11.826152108	-9.428716765	1.356679163
1	51	7.709241044	14.925552797	-2.751728394
1	51	7.327700283	10.718384313	0.772751699
1	51	3.724893935	8.331615255	-2.642553318
1	51	3.654112613	3.930319510	0.622493644
1	51	-0.099037501	1.739726018	-2.742184157
1	51	0.007507237	-2.829088770	0.293282431

1	51	-4.118216087	-5.025684180	-2.571311725
1	51	-3.780910473	-9.525392766	0.557647159
1	51	-8.103538577	-11.760662837	-1.981660831
1	51	-7.800698324	-16.473746186	0.828929684
1	51	11.651492497	8.467650095	-1.745216187
1	51	11.682429356	3.769281179	1.097515853
1	51	7.810996258	1.813851835	-2.287716124
1	51	7.697269723	-3.089643245	0.191164882
1	51	3.747334576	-4.943288329	-3.147263505
1	51	3.875134319	-9.677616913	-0.352242171
1	51	-0.372414268	-11.791083753	-3.125028510
1	51	-0.059932343	-16.361117879	-0.080950763
1	51	15.523525005	1.721662989	-2.268376199
1	51	15.440508196	-3.094260362	0.371807410
1	51	11.641591498	-4.998583331	-3.119718627
1	51	11.433112004	-9.740546994	-0.359374941
1	51	7.636198117	-11.890954882	-3.708435754
1	51	7.421757747	-16.262482832	-0.382234510
1	51	-15.097291392	20.329454618	6.263921188
1	51	-14.362163784	16.201223642	9.897250006
1	51	-17.568222763	13.143863665	6.659006588
1	51	-16.495996367	8.573844415	9.495312275
1	51	-20.096728019	5.779935456	6.451355841
1	51	-18.760241301	1.336430421	9.365253943
1	51	-7.456651767	22.169183888	7.450461165
1	51	-6.688172608	17.861575314	10.768248176
1	51	-9.716355320	14.872469609	7.300615193
1	51	-8.990581379	10.305838188	10.216482992
1	51	-12.002391809	7.591508721	6.482512949
1	51	-11.275667026	3.164607925	9.635085054
1	51	-14.374201157	0.151351151	6.257090582
1	51	-13.469021756	-4.450290820	9.128228193
1	51	-0.208411379	24.124986053	7.465310012
1	51	1.150438791	19.700994160	10.444412376
1	51	-2.426254033	16.742747822	7.490275343
1	51	-1.176938681	12.135899697	10.182654983
1	51	-4.623579042	9.167718024	7.083928182
1	51	-3.706683932	4.750761357	10.206905618
1	51	-6.788556263	1.635823887	6.902325140
1	51	-6.074474035	-2.742753068	10.167700889
1	51	-8.847302049	-5.843863540	6.561575267
1	51	-8.386327523	-10.313700415	9.713025908
1	51	4.971001599	18.526210445	6.664471521
1	51	6.491005658	14.002897418	9.371533310
1	51	2.799680400	11.029356025	6.546475488
1	51	3.985433385	6.636313571	9.591256514
1	51	0.492077708	3.280992882	7.016679276
1	51	1.667857900	-0.847104437	10.450573572
1	51	-1.531174930	-4.115385775	7.408500987
1	51	-0.579424678	-8.419428874	10.695358082
1	51	10.598373133	12.952510216	5.814040100
1	51	11.529287428	8.441959281	8.810473368

1	51	8.016136073	5.178948442	6.150044717
1	51	9.267196324	1.116007707	9.683669054
1	51	5.786690111	-2.267573685	7.125608348
1	51	7.233657347	-6.559919024	10.213522122
2	6	16.834883076	-6.809351662	16.313504075
2	6	14.314816761	-5.814643844	16.223482623
2	6	12.262861525	-7.586074727	16.266305099
2	6	12.700567177	-10.172093001	16.174755703
2	6	15.223867202	-11.162120815	16.045303108
2	6	17.274605558	-9.395496157	16.236145845
2	6	15.680741608	-13.814303696	15.623115672
2	6	13.860405906	-3.135977161	15.968383760
2	6	18.163207581	-14.759227399	15.207755612
2	6	15.873038205	-1.404536929	15.571483122
2	6	13.656598714	-15.542838834	15.221229950
2	6	11.388965352	-2.166644398	15.560428583
3	7	11.946808794	-16.946177311	14.831359773
3	7	17.545247713	0.030194508	15.118546856
4	1	11.091934423	-11.499724133	16.166317124
4	1	18.444208669	-5.484048388	16.403126046
3	7	20.223970866	-15.528832579	14.753091295
3	7	9.337245022	-1.373137038	15.095808846
4	1	10.306182815	-6.864122971	16.325399952
4	1	19.229449817	-10.120564371	16.289451206

9.43º

		-8.579431412	47.547946397	0.000000000
		-44.175927830	15.605524497	0.000000000
		0.000000000	0.000000000	110.891359767
		148		
1	51	-10.532750498	22.971823956	-1.108990965
1	51	-11.541983875	18.036150345	1.190123295
1	51	-15.066393248	16.042901402	-2.761133270
1	51	-15.563076096	11.400685973	0.068590824
1	51	-17.896406619	9.260319164	-4.421469391
1	51	-19.561828836	5.054269528	-1.304155115
1	51	-22.534009476	2.345893298	-5.057216765
1	51	-23.977851555	-0.325863458	-0.470031448
1	51	-2.506500243	22.295486118	-2.476019320
1	51	-3.323937006	17.772797769	0.586861210
1	51	-7.726287143	15.804542828	-2.141830797
1	51	-7.426249436	11.345161676	1.099377615
1	51	-11.182133608	9.106599269	-2.188621503
1	51	-11.951978711	4.718245726	1.045646657
1	51	-15.194463173	2.111242592	-2.574194986
1	51	-16.785139760	-1.652818805	1.120946252
1	51	-20.557555790	-4.370851531	-2.008895878
1	51	-21.057761755	-8.104651109	2.012684690
1	51	5.644386509	21.234760783	-2.596745535
1	51	3.834153403	17.231822626	0.685146034
1	51	0.113659295	15.131342216	-2.751402692
1	51	0.155919360	10.870757095	0.690734033

1	51	-3.922577734	8.811470436	-2.273638742
1	51	-3.797320811	4.374864170	0.939585046
1	51	-8.018405835	2.222719067	-1.803016864
1	51	-8.277275598	-1.992856104	1.652807551
1	51	-12.488380834	-4.336289445	-1.049807513
1	51	-12.804623036	-8.417431698	2.552879382
1	51	-16.819687567	-10.652100971	-0.554376262
1	51	-16.485059178	-15.234020813	2.459711836
1	51	9.301521515	21.655419517	1.605162011
1	51	11.203643976	16.466881073	2.283861683
1	51	7.931566128	14.373011919	-1.497141843
1	51	7.645755512	9.780786505	1.397121702
1	51	3.918476187	8.291274519	-2.335188197
1	51	3.694160593	3.674577486	0.513506582
1	51	-0.421369372	1.961215601	-2.668524802
1	51	-0.347504661	-2.452133576	0.519259967
1	51	-4.837548975	-4.526230480	-1.830757276
1	51	-4.375951658	-8.596133800	1.771561013
1	51	-8.842064200	-10.818637267	-0.483797612
1	51	-8.557176159	-15.161736874	2.793530680
1	51	-12.326199630	-17.349055396	-0.481927551
1	51	-11.496014862	-22.048273741	2.142981824
1	51	15.363348222	14.600588023	-0.854570513
1	51	15.753951704	9.745681647	1.744639673
1	51	11.828882479	7.838192042	-1.572083454
1	51	11.866360877	2.885464640	0.681535420
1	51	7.663953088	1.710959649	-2.755295543
1	51	7.390407829	-3.122106123	-0.295099685
1	51	3.018020916	-4.899051104	-3.079955818
1	51	3.353914080	-9.189495216	0.302278330
1	51	-1.212965466	-11.278542191	-1.855256290
1	51	-0.444794821	-15.379605333	1.693439211
1	51	-4.912265088	-17.673371731	-0.463640959
1	51	-4.968031566	-22.042126640	2.831458738
1	51	20.098495774	7.992155917	-1.181031725
1	51	20.152229578	2.826998544	0.667754558
1	51	15.942300829	1.647271665	-2.853371001
1	51	15.303183565	-3.680573302	-1.654502558
1	51	10.635848823	-4.975988662	-4.275974583
1	51	10.959485019	-9.576706019	-1.265136971
1	51	6.468430999	-11.968843603	-3.279062620
1	51	7.321359071	-16.220481417	0.154631779
1	51	2.595504867	-18.299246695	-1.865342348
1	51	2.770689001	-22.845247020	1.238115881
1	51	23.567380654	1.338949160	-3.498302201
1	51	22.025532521	-3.791085609	-2.243575306
1	51	18.282974682	-5.149620264	-6.046330872
1	51	18.160586915	-9.644704005	-2.877653655
1	51	13.908454695	-12.431532531	-4.861658952
1	51	14.478593408	-15.823555844	-0.578453935
1	51	10.748107268	-18.961721543	-3.253977043
1	51	10.547257900	-23.255379254	0.218325562

1	51	-16.874909697	27.884308827	7.447659683
1	51	-17.183244941	23.019420142	9.970126809
1	51	-20.253623026	20.541534113	6.108579627
1	51	-20.356213406	15.704841046	8.821230526
1	51	-23.349848937	13.206567156	4.932082037
1	51	-23.312452034	8.492137821	7.895084224
1	51	-26.772028839	5.629504368	4.670617286
1	51	-26.558705179	1.687571525	8.549298532
1	51	-9.326983765	27.632955009	6.008186788
1	51	-9.280251789	24.064279201	10.190992510
1	51	-12.799938266	20.989612216	7.437557109
1	51	-12.659687325	16.511585017	10.596270280
1	51	-15.591907963	14.196709587	6.562528133
1	51	-15.826691541	9.511681253	9.386131059
1	51	-18.647325869	6.804717025	5.502228427
1	51	-18.705673481	2.490169059	8.932356204
1	51	-22.199508872	-0.747322459	6.143992482
1	51	-21.539097031	-4.455435344	10.172262041
1	51	-3.010136780	29.612848861	7.152470012
1	51	-1.769960501	25.374499318	10.533769132
1	51	-4.937919875	21.858947482	7.751905786
1	51	-4.637092133	17.700455993	11.304653618
1	51	-8.103005523	15.147060618	7.870389020
1	51	-7.880222072	10.556583074	10.832549363
1	51	-11.055928612	7.991217344	7.141687722
1	51	-11.089263126	3.511001226	10.288548922
1	51	-14.051187842	0.541591230	6.712104685
1	51	-13.770110808	-3.815905557	10.090157670
1	51	-17.469746133	-6.492767623	7.035191176
1	51	-16.974615576	-10.991848561	10.136253011
1	51	5.276921809	30.247289368	6.288746685
1	51	5.956822620	26.533801524	10.326458064
1	51	2.401519400	23.373901605	7.554935440
1	51	3.050490214	18.962124709	10.829805757
1	51	-0.628518036	16.009050390	7.996945449
1	51	0.055751345	11.432236138	10.945144881
1	51	-3.650633476	8.852124945	7.805223923
1	51	-3.124305855	4.305008406	10.837247178
1	51	-6.701984873	1.647278885	7.615268132
1	51	-6.223714107	-2.843531940	10.746771125
1	51	-9.434697370	-5.896656272	7.448756457
1	51	-9.015807814	-10.605450482	10.393760462
1	51	-12.784681963	-13.059577223	7.220119025
1	51	-12.686344834	-17.815996212	10.023806246
1	51	10.221136573	24.667205330	7.370924714
1	51	10.745408572	19.974553902	10.192870695
1	51	6.943041397	17.291842108	7.254695114
1	51	7.696894037	12.530271337	9.956092206
1	51	3.749012517	9.893267239	7.198365879
1	51	4.677036862	5.174683414	9.863433712
1	51	0.638808311	2.531583155	7.272999162
1	51	1.672456582	-1.940915332	10.296297660

1	51	-2.229593904	-4.511697089	7.435470892
1	51	-1.659529235	-9.027443051	10.484250748
1	51	-4.752708883	-12.255259523	7.292099842
1	51	-2.952603365	-16.804752157	9.943619294
1	51	14.942267683	18.303316900	6.988370679
1	51	15.370206010	13.433004486	9.508117991
1	51	11.595962472	11.051670072	6.357988769
1	51	12.135459522	6.092736012	8.790156846
1	51	8.180688048	3.641157123	5.909020539
1	51	9.219659695	-1.151561952	8.474745647
1	51	4.972441633	-3.875401651	6.366501900
1	51	6.285649241	-8.162536591	9.569725920
1	51	2.259098453	-11.108320659	7.342692288
1	51	2.563035704	-15.202962301	10.940831752
1	51	19.627795370	11.605475623	6.393646815
1	51	19.595193433	6.668293800	8.909784790
1	51	16.068966472	4.331844213	5.381459056
1	51	16.470375016	-0.682568010	7.797671095
1	51	12.667754255	-3.185676701	4.707675546
1	51	13.480792234	-7.753012164	7.827800692
1	51	9.297467780	-10.641183352	5.710069983
1	51	9.851806854	-14.880451060	9.226005419

6.01°

-3.895200590	74.586629276	0.094623857
-66.525662320	33.886886771	-0.025077985
0.039257065	0.049437513	48.137238661
364		

1	51	-19.263643521	36.162618666	-0.556268580
1	51	-19.142301184	31.451572493	2.313800092
1	51	-23.060628167	29.219357702	-0.853817255
1	51	-23.144682685	24.379162031	1.756008874
1	51	-27.041257048	22.415921851	-1.600098698
1	51	-27.250039590	17.689751756	1.192569715
1	51	-31.103452516	15.647307549	-2.153747191
1	51	-31.281644769	11.065868777	0.866936090
1	51	-35.131498703	8.923688058	-2.416122531
1	51	-35.278258596	4.387727542	0.668775330
1	51	-39.173828579	2.201901078	-2.528161645
1	51	-39.266002028	-2.278292520	0.640844799
1	51	-11.585553511	36.388104488	-0.874081433
1	51	-11.374667591	31.613918712	1.896755934
1	51	-15.391900080	29.362077282	-1.101532836
1	51	-15.400243497	24.554867651	1.611459365
1	51	-19.303272790	22.358409695	-1.584193772
1	51	-19.455003698	17.726612039	1.384854660
1	51	-23.280116561	15.509329261	-1.886886343
1	51	-23.485502653	10.981092481	1.215197775
1	51	-27.276628675	8.805980116	-2.120858795
1	51	-27.495346774	4.269324948	0.952170696
1	51	-31.311953066	2.106237588	-2.361449587
1	51	-31.481485557	-2.386023676	0.775084114

1	51	-35.353788895	-4.639848781	-2.404182960
1	51	-35.489388679	-9.036980354	0.876623500
1	51	-3.978520266	36.417296182	-1.582134826
1	51	-3.813086517	31.762714740	1.386810600
1	51	-7.772086854	29.431750781	-1.615051487
1	51	-7.786773838	24.804277511	1.405792540
1	51	-11.627458731	22.392659429	-1.700766870
1	51	-11.724990020	17.894418177	1.489219244
1	51	-15.523523607	15.469724167	-1.665583242
1	51	-15.660470612	10.997529978	1.528271585
1	51	-19.462236055	8.725971266	-1.737805040
1	51	-19.645412660	4.190200453	1.331647131
1	51	-23.454818972	2.060746629	-2.027672835
1	51	-23.642238204	-2.472971408	1.028165963
1	51	-27.500029060	-4.694264962	-2.215029608
1	51	-27.685817251	-9.057922952	1.088504809
1	51	-31.524694080	-11.509977940	-2.004572127
1	51	-31.809837026	-15.770089598	1.458743844
1	51	3.800079773	36.350524320	-2.087896908
1	51	3.815885850	31.886485464	1.138364893
1	51	-0.076596277	29.482555281	-1.889969814
1	51	-0.134415430	25.018753876	1.358242735
1	51	-3.936394114	22.524582634	-1.720785330
1	51	-3.999338188	18.086066318	1.552052133
1	51	-7.803863076	15.611340253	-1.549407189
1	51	-7.844834950	11.141079253	1.655780211
1	51	-11.681701374	8.796388582	-1.517672485
1	51	-11.749652823	4.269844672	1.572535321
1	51	-15.597681958	2.091350205	-1.714825244
1	51	-15.729042069	-2.465767247	1.295922824
1	51	-19.601986351	-4.635762945	-1.987778168
1	51	-19.750171045	-9.022816092	1.271840817
1	51	-23.665806938	-11.483898919	-1.743212133
1	51	-23.926507379	-15.672519188	1.815249299
1	51	-27.730591948	-18.323247895	-1.166069497
1	51	-27.931167036	-22.655759903	2.247539524
1	51	11.612866218	36.250956524	-2.260942063
1	51	11.583929665	31.857753901	1.044576215
1	51	7.645111317	29.473425756	-1.938440902
1	51	7.596992940	25.089952886	1.395548422
1	51	3.755428718	22.643482547	-1.661010391
1	51	3.721295133	18.203831476	1.602489904
1	51	-0.095727424	15.787367570	-1.511520104
1	51	-0.065106226	11.306685097	1.679055630
1	51	-3.907598430	8.952002278	-1.461562169
1	51	-3.875299007	4.443632946	1.663108473
1	51	-7.739943865	2.194521646	-1.543667715
1	51	-7.779686780	-2.345900797	1.501318012
1	51	-11.659316145	-4.507258389	-1.772565698
1	51	-11.738514373	-8.975639502	1.358240409
1	51	-15.725652496	-11.288154455	-1.688949092
1	51	-15.857526222	-15.487759089	1.833431345

1	51	-19.855636403	-18.067715418	-0.970241939
1	51	-19.926038421	-22.441394157	2.364559091
1	51	-23.838744327	-24.919531586	-0.633915746
1	51	-23.668103428	-29.504869661	2.411872501
1	51	19.435404793	36.149056808	-2.327995381
1	51	19.391195697	31.745928344	0.955605548
1	51	15.420871532	29.402496473	-2.016190969
1	51	15.410797095	25.028234121	1.316474782
1	51	11.458097044	22.680508933	-1.661757085
1	51	11.502088231	18.231474276	1.579467771
1	51	7.617111383	15.900435324	-1.492580918
1	51	7.720215416	11.399848799	1.668604691
1	51	3.855603193	9.098542092	-1.459126325
1	51	3.970321888	4.587759684	1.671016431
1	51	0.095047914	2.310954331	-1.476258921
1	51	0.153851589	-2.201302246	1.626170713
1	51	-3.729072848	-4.392253245	-1.598628026
1	51	-3.776357218	-8.921903819	1.448792959
1	51	-7.721803618	-11.090140587	-1.738193640
1	51	-7.728219927	-15.425122023	1.592153218
1	51	-11.877108756	-17.837338832	-1.133681495
1	51	-11.839384490	-22.142929030	2.267280341
1	51	-15.840999586	-24.598624286	-0.628329194
1	51	-15.700516626	-29.183558625	2.406568970
1	51	-19.593658462	-31.643149187	-0.626640155
1	51	-19.397441713	-36.251490128	2.396479462
1	51	23.259887054	29.297791242	-2.081907747
1	51	23.133160660	24.944797659	1.270097802
1	51	19.240636594	22.593022933	-1.782061103
1	51	19.243713265	18.153577980	1.468328145
1	51	15.328860525	15.898691592	-1.606428537
1	51	15.511897371	11.376792513	1.521249003
1	51	11.578493403	9.138966138	-1.535632009
1	51	11.811659252	4.603326456	1.558915809
1	51	7.873754619	2.382236026	-1.517410573
1	51	8.051071295	-2.147663243	1.574388986
1	51	4.108387002	-4.339707045	-1.542763300
1	51	4.161175211	-8.893416533	1.492394932
1	51	0.217733333	-11.008978738	-1.703416260
1	51	0.222664566	-15.532101192	1.361668272
1	51	-3.875544118	-17.727595169	-1.587053227
1	51	-3.718486764	-22.065284046	1.757375735
1	51	-7.928429503	-24.417466299	-0.880455754
1	51	-7.661772839	-28.907146999	2.282162257
1	51	-11.644328921	-31.333221474	-0.630375386
1	51	-11.463621192	-35.971922055	2.354900474
1	51	27.093285703	22.402353154	-1.564580504
1	51	26.852598026	18.050397712	1.803953518
1	51	23.175915184	15.705210091	-1.507949321
1	51	23.117947451	11.216671874	1.678183107
1	51	19.371312538	9.044355968	-1.656067018
1	51	19.519988700	4.504937007	1.441632176

1	51	15.630198241	2.327483230	-1.708157682
1	51	15.877501285	-2.223270648	1.355607088
1	51	11.885795501	-4.377233219	-1.694125661
1	51	12.051436025	-8.948772215	1.331279910
1	51	8.040742799	-11.052011674	-1.759470994
1	51	8.075357052	-15.661613007	1.197468226
1	51	4.051048734	-17.756251373	-1.900995715
1	51	4.135532432	-22.276296867	1.195423008
1	51	-0.063993973	-24.472840374	-1.585983628
1	51	0.257973444	-28.958257452	1.570029098
1	51	-3.966887730	-31.235974186	-1.084678440
1	51	-3.609989069	-35.877265630	1.871690927
1	51	30.983485531	15.540927750	-0.812127522
1	51	30.948081146	11.035781256	2.370926657
1	51	27.273823107	8.804687513	-1.033393062
1	51	27.217785607	4.196196577	1.963061280
1	51	23.534112248	2.185452422	-1.564237323
1	51	23.556792638	-2.430578257	1.405086054
1	51	19.715426785	-4.496679707	-1.894380767
1	51	19.819322238	-9.089671510	1.107081543
1	51	15.827868583	-11.177442178	-2.000935624
1	51	15.890213724	-15.792925394	0.961255187
1	51	11.857884075	-17.869504633	-2.126317738
1	51	11.895651142	-22.465828845	0.865905196
1	51	7.828592571	-24.602822851	-2.145954817
1	51	7.944245233	-29.138777218	0.947376925
1	51	3.798484044	-31.335547256	-1.902332370
1	51	4.029665129	-35.907093194	1.159453622
1	51	35.071027978	8.777138492	-0.508339048
1	51	35.257336273	4.158262255	2.484834925
1	51	31.443630701	1.996682213	-0.827919010
1	51	31.427105216	-2.713148058	1.988356821
1	51	27.674900949	-4.603560819	-1.562947888
1	51	27.562769792	-9.313168935	1.242808318
1	51	23.707790395	-11.295886057	-2.110236314
1	51	23.652092729	-15.924448954	0.837057539
1	51	19.691750140	-18.005163741	-2.335557427
1	51	19.677096791	-22.585882703	0.686885993
1	51	15.681557747	-24.729558674	-2.410091916
1	51	15.681645713	-29.266869327	0.678664844
1	51	11.664237003	-31.458270779	-2.355914754
1	51	11.701072058	-35.959239245	0.797295428
1	51	39.349989526	2.007130973	-0.526252896
1	51	39.460345406	-2.670378079	2.360807023
1	51	35.600504166	-4.704182524	-1.010103715
1	51	35.461105331	-9.518257945	1.611394346
1	51	31.618884486	-11.382441665	-1.850635459
1	51	31.442178981	-16.083577265	0.976941905
1	51	27.565987982	-18.128793431	-2.327685186
1	51	27.457605326	-22.708026563	0.698125192
1	51	23.539000838	-24.853804090	-2.492800392
1	51	23.464329385	-29.379171872	0.613768132

1	51	19.514508439	-31.576430580	-2.504385123
1	51	19.474207343	-36.053743362	0.674487148
1	51	-27.304892962	40.391140872	6.958204294
1	51	-26.990857610	35.810890118	9.994896546
1	51	-30.825020922	33.154627499	7.086958907
1	51	-30.203487136	28.830782677	10.452913943
1	51	-34.000440418	25.908578773	7.787406280
1	51	-33.262252806	21.693842151	11.240059064
1	51	-36.960807430	18.795774772	8.398249578
1	51	-36.351348836	14.451829787	11.697444986
1	51	-39.989744398	11.684105761	8.655236768
1	51	-39.429045044	7.234504911	11.823443585
1	51	-43.044538140	4.522019055	8.705692883
1	51	-42.512665730	0.024699697	11.812311959
1	51	-19.345864942	40.894418171	6.960311082
1	51	-19.003446134	36.329621204	10.007754038
1	51	-22.832427094	33.759409774	7.002257482
1	51	-22.333334825	29.408971053	10.325444661
1	51	-26.113057945	26.623979146	7.485070776
1	51	-25.444871140	22.470439323	11.029966598
1	51	-29.171920005	19.631672261	8.180899833
1	51	-28.554170632	15.329650858	11.520355715
1	51	-32.259213353	12.591419962	8.536180362
1	51	-31.630965631	8.140234887	11.678150212
1	51	-35.326149739	5.452328003	8.637096014
1	51	-34.719009818	0.954712441	11.727331775
1	51	-38.386664878	-1.719465147	8.639813831
1	51	-37.822814351	-6.243972167	11.700026407
1	51	-11.305010944	41.449045754	7.110659015
1	51	-11.114074126	36.984740711	10.308375214
1	51	-14.746041986	34.307576709	7.140887499
1	51	-14.365906717	30.034781093	10.557953295
1	51	-18.052585983	27.275389476	7.559086367
1	51	-17.516443111	23.096947074	11.069244036
1	51	-21.254202214	20.423061210	8.069576058
1	51	-20.693935708	16.079720265	11.343486218
1	51	-24.468517495	13.497388222	8.311253679
1	51	-23.829780300	9.010599946	11.380543056
1	51	-27.597909070	6.382702876	8.382232927
1	51	-26.923985698	1.880298344	11.445425538
1	51	-30.667260685	-0.787579832	8.452800690
1	51	-30.018062765	-5.322956209	11.484753514
1	51	-33.758596548	-7.917715738	8.416687610
1	51	-33.101451351	-12.541473623	11.302737038
1	51	-3.425000695	42.320624153	7.868419817
1	51	-3.277007401	37.850458693	11.055469002
1	51	-6.659538730	35.085163438	7.704484448
1	51	-6.379149356	30.753016047	11.043214164
1	51	-9.973399514	28.082161940	7.844060559
1	51	-9.537182520	23.753445013	11.151736480
1	51	-13.294667069	21.225399102	8.037509890
1	51	-12.773888156	16.782955390	11.165156998

1	51	-16.598096701	14.322172371	8.086127070
1	51	-16.004943296	9.801147458	11.102342173
1	51	-19.800302386	7.279738051	8.048986810
1	51	-19.168481847	2.770420322	11.103925607
1	51	-22.917653879	0.121399716	8.115211742
1	51	-22.266258810	-4.378449139	11.200948261
1	51	-25.987859023	-6.979220991	8.135803854
1	51	-25.286349951	-11.636405818	10.966329352
1	51	-29.108969733	-14.021200840	7.827179217
1	51	-28.428822855	-18.780056141	10.463347054
1	51	4.196025928	43.327634827	8.493814806
1	51	4.554564889	38.810511469	11.591734023
1	51	1.117863960	36.121994371	8.248952546
1	51	1.507634753	31.608392736	11.325739777
1	51	-2.086592019	29.015689904	8.066453578
1	51	-1.622030789	24.503114331	11.110181078
1	51	-5.386405077	22.019993818	7.960622171
1	51	-4.887209378	17.509674366	11.000496652
1	51	-8.689532521	15.034282280	7.897364273
1	51	-8.188323624	10.525695205	10.953864404
1	51	-11.943636523	8.023393549	7.832164680
1	51	-11.423860313	3.526673871	10.926257153
1	51	-15.130622027	0.918701233	7.855545646
1	51	-14.560213640	-3.533030537	11.033242687
1	51	-18.211582874	-6.187582418	7.953626281
1	51	-17.584968904	-10.774457050	10.932645340
1	51	-21.206798178	-13.190404399	7.608587398
1	51	-20.602626148	-17.985403231	10.226181498
1	51	-24.396831280	-20.366103824	7.046297825
1	51	-23.992076438	-25.028711964	9.924360809
1	51	11.868217947	44.280196201	8.756312672
1	51	12.335876474	39.763911309	11.841471814
1	51	8.809560578	37.122906685	8.559073669
1	51	9.248271152	32.536663907	11.535112504
1	51	5.713831515	29.951485701	8.208194855
1	51	6.136555899	25.358362505	11.160610975
1	51	2.508425106	22.804600612	7.903700479
1	51	2.912022895	18.280251032	10.955949189
1	51	-0.792682147	15.723319324	7.792147835
1	51	-0.390580974	11.238022534	10.912862300
1	51	-4.099532004	8.681273348	7.769771176
1	51	-3.681127703	4.192399150	10.897267752
1	51	-7.354772858	1.600066287	7.767448413
1	51	-6.869008920	-2.845448866	10.972215598
1	51	-10.500876341	-5.530952979	7.903251784
1	51	-9.938753421	-10.012595760	11.071470712
1	51	-13.460573824	-12.547261851	7.746134315
1	51	-12.939675613	-17.331919809	10.446249012
1	51	-16.473681316	-19.785934773	7.044930644
1	51	-16.087208740	-24.477203868	9.916239917
1	51	-19.876224544	-27.047241996	6.872207437
1	51	-19.577944884	-31.646602221	9.898555931

1	51	16.530998595	38.054876901	8.742338487
1	51	16.999221324	33.507917185	11.779042354
1	51	13.481557139	30.889265446	8.463122673
1	51	13.848287626	26.301703973	11.446831911
1	51	10.353990417	23.683092380	8.089357637
1	51	10.622100797	19.134899497	11.140833608
1	51	7.067351993	16.497004755	7.858129002
1	51	7.343370657	11.987470315	10.970546099
1	51	3.704291471	9.373406818	7.776520637
1	51	4.045648065	4.876925176	10.910602013
1	51	0.391761437	2.261513084	7.760082993
1	51	0.820739218	-2.205832582	10.940860435
1	51	-2.821568309	-4.892537926	7.869427511
1	51	-2.292243830	-9.317763495	11.120862219
1	51	-5.856795580	-11.989685049	7.965007499
1	51	-5.374006649	-16.605919132	10.963800846
1	51	-8.713207649	-19.149729404	7.436882177
1	51	-8.409817219	-23.912997142	10.215576235
1	51	-11.946359687	-26.516712235	6.910338591
1	51	-11.638774204	-31.139305479	9.906936351
1	51	21.172203159	31.823859678	8.650149104
1	51	21.662198923	27.252675404	11.647348949
1	51	18.071418777	24.628943424	8.399015791
1	51	18.398779222	20.062254596	11.422115098
1	51	14.845376269	17.401747326	8.137710334
1	51	15.046053652	12.846441315	11.198927162
1	51	11.502075031	10.184052451	7.914413858
1	51	11.725465676	5.650994449	11.015027413
1	51	8.138437652	2.997002501	7.795805143
1	51	8.481896225	-1.496484905	10.951075585
1	51	4.874922528	-4.206034689	7.833646403
1	51	5.362513900	-8.614653474	11.101998587
1	51	1.768899950	-11.383794039	8.054215755
1	51	2.268872077	-15.822835645	11.298147828
1	51	-1.168028348	-18.500438653	7.990153667
1	51	-0.848423644	-23.149076704	10.953970100
1	51	-4.098194975	-25.810165712	7.398430261
1	51	-3.987838027	-30.437418853	10.390696205
1	51	25.721779609	25.651966417	8.336922963
1	51	26.306367781	20.992617356	11.166636949
1	51	22.460922673	18.410466161	8.159219340
1	51	22.889358446	13.809181275	11.116780644
1	51	19.159153927	11.098467054	8.070403393
1	51	19.460971900	6.563928559	11.160186465
1	51	15.877139370	3.846266395	7.964680291
1	51	16.152873687	-0.662531172	11.099984091
1	51	12.610348767	-3.397601830	7.902641181
1	51	13.009391352	-7.819177690	11.153158120
1	51	9.458330376	-10.631967373	8.064769674
1	51	9.960970545	-14.985929284	11.398540891
1	51	6.423715293	-17.786826338	8.299630400
1	51	6.871967659	-22.243923228	11.505631959

1	51	3.499814315	-24.959336446	8.146893396
1	51	3.731807307	-29.509983085	11.237335519
1	51	30.233392149	19.522322587	7.604257421
1	51	30.761137566	14.829679887	10.391470826
1	51	26.779203751	12.224270518	7.590865205
1	51	27.319059515	7.627280614	10.549378475
1	51	23.425633827	4.821263909	7.825065133
1	51	23.957499451	0.356663943	10.993714135
1	51	20.289293509	-2.485784403	8.029575830
1	51	20.753678502	-6.898741079	11.279482735
1	51	17.198626863	-9.728984246	8.188814104
1	51	17.678867857	-14.085759017	11.508814569
1	51	14.140204772	-16.919800862	8.418821428
1	51	14.626806739	-21.304326096	11.699409451
1	51	11.106416110	-24.073344140	8.534799260
1	51	11.540894785	-28.546565485	11.697022294
1	51	34.777248347	13.109621142	7.048680747
1	51	35.093852521	8.492694719	10.027304400
1	51	31.220942271	5.837010158	7.133098505
1	51	31.715269990	1.340422632	10.280228387
1	51	27.851447175	-1.554228306	7.647249062
1	51	28.558637689	-5.876678874	10.983167493
1	51	24.851551944	-8.816504666	8.189609134
1	51	25.476672908	-13.139397879	11.526991759
1	51	21.865163570	-15.977629349	8.511661193
1	51	22.388021732	-20.368743113	11.772552032
1	51	18.812217546	-23.143694264	8.665011281
1	51	19.322188995	-27.593568633	11.844768592
